# Correlation analysis of Blood TM, TG and D-dimer with deep venous Thrombosis formation in patients after Total Hip Arthroplasty

**DOI:** 10.12669/pjms.39.2.6989

**Published:** 2023

**Authors:** Hailin Luo, Yong Qiao

**Affiliations:** 1 Hailin Luo, Department of Vascular Surgery, Sichuan Province Orthopedics Hospital, Chengdu 610041, Sichuan, China; 2 Yong Qiao, Department of Vascular Surgery, Sichuan Province Orthopedics Hospital, Chengdu 610041, Sichuan, China

**Keywords:** Thrombomodulin, Triglycerides, D-dimer, Total hip arthroplasty, Deep venous thrombosis

## Abstract

**Objective:**

To explore the correlation of blood thrombomodulin (TM), triglyceride (TG) and D-Dimer (D-D) with the formation of deep vein thrombosis (DVT) in patients after total hip arthroplasty (THA).

**Methods:**

A retrospective analysis was carried out focusing on the clinical data of 150 patients with THA who were admitted to Sichuan Provincial Orthopedic Hospital from May 2019 to May 2022 (the study group). These patients were then subdivided into Group-A (46 cases with DVT) and Group-B (104 cases without DVT) according to whether DVT occurred after an operation. Meanwhile, another 70 patients who received physical examination in this hospital in the same period were selected as the control group. Furthermore, the levels of TM, TG, D-D, fibrinogen (Fb) and C-reactive protein (CRP) were compared between the groups. Pearson correlation was used to analyze the relationship between the levels of TM, TG, D-D, Fb, CRP and the formation of DVT.

**Result:**

The levels of TM, TG, D-D, Fb and CRP in the study group were significantly higher than those in the control group (p<0.05). The above indicators in Group-A were higher than those in Group-B one day after the operation (p<0.05). Pearson correlation analysis showed that the levels of TM, TG, D-D, Fb and CRP were significantly positively correlated with the formation of DVT.

**Conclusion:**

Patients with DVT after THA show an increase in the levels of TM, TG, D-D, Fb and CRP, indicating their diagnostic value for the formation of DVT. Findings in our study suggest that clinical tests of these indicators can be carried out according to the actual situation of patients.

## INTRODUCTION

Artificial joint replacement is an effective surgical option that is commonly applied for patients with severe joint injuries. Total hip arthroplasty (THA) can restore the function of the hip joint in patients to the greatest extent by implanting a prosthesis, which is an ideal lower limb surgery.^1^ However, there is a need for long-term stay in bed for patients undergoing THA, leading to a relatively higher risk of DVT formation.^2^ According to previous studies, DVT formation may occur in over 30% of patients undergoing THA postoperatively, which is generally related to factors such as peripheral vascular injury and vessel intimal injury caused by surgery.^3^

In the absence of effective control, pulmonary embolism will occur in patients with serious progress, which may be life-threatening. DVT is characterized by occult onset and low diagnostic rate using conventional Doppler ultrasound, resulting in the lack of effective control of the occurrence of DVT.^4^

Meanwhile, venography is costly and time-consuming, despite high diagnostic accuracy.^5^ Consequently, it is important to determine a more convenient and efficient diagnostic mode. Thrombomodulin (TM) is a transmembrane single-chain glycoprotein, which is related to endothelial cell injury. Its expression may increase significantly when there is vascular damage.^6^ Triglyceride (TG) is a routine indicator of blood lipid detection that can reveal atherosclerosis in patients. The elevation of TG level has been reported to be related to the formation of DVT.^7^ In addition, D-Dimer (D-D) is a special marker of secondary fibrinolysis, which can indicate the presence of thrombosis. Therefore, this study analyzed and compared the clinical data of 150 patients, with the purpose to seek the indicators related to the formation of DVT, so as to provide scientific reference for the early prevention and treatment of patients.

## METHODS

A retrospective analysis was carried out focusing on the clinical data of 150 patients with THA who were admitted to Sichuan Province Orthopedic Hospital from May 2019 to May 2022 (the study group). The study was approved by the Institutional Ethics Committee of Sichuan Province Orthopedics Hospital (No.: 2017-6-30-1; Date: June 30, 2017).

### Inclusion criteria:

- Patients who meet the surgical indications of THA and had unilateral surgery;
- Patients with complete clinical data;
- Patients who met the ethical review criteria in the Declaration of Helsinki;
- Patients without a history of the thrombotic disease;
- Patients without cognitive impairment;
- Patients without pregnancy;
- Patients without malignant tumor disease.

### Exclusion criteria:

- Patients who met the diagnostic and therapeutic criteria of acute ischemic infarction
- Patients with mental illness;
- Patients with dysfunction of important organs;
- Patients with postoperative infection;
- Patients with related treatment of anti-coagulation before and after surgery.

Among the 150 patients in the study group, there were 101 males and 49 females, with an average age of (50.25±10.31) years (35~69 years old). There were 51 cases of femoral head necrosis and 99 cases of a traumatic femoral neck fracture. Meanwhile, another 70 patients who received physical examination in Sichuan Provincial Orthopedic Hospital in the same period were selected as the control group. There were 46 males and 24 females, with an average age of (50.71±9.99) years (32~70 years old). There was no significant difference in gender and age between the two groups (p>0.05), suggesting the existence of comparability between the two groups.

An amount of 5 ml of fasting cubital venous blood from each patient was collected at different time points in the morning. The blood samples were then subject to centrifugation at 3500 r/min for 10 minutes, the upper serum was separated and stored in a low-temperature environment for testing. Beckman Uni Cel Dx I800 automatic analyzer (USA) was used to analyze TG level, chemiluminescence to detect TM level, immunological turbidimetry assay to test D-D and CRP levels, and coagulation method to measure FB level. All tests were carried out by the same group of medical staff in strict accordance with the standards described in the manual under the premise of quality control.

Ultrasonic diagnostic criteria of DVT were described as follows:^8^ (1) the presence of parenchymal echo in the lumen; (2) no compression of the venous vessels; (3) the lack of autonomous blood flow at the site of thrombus indicated by color and pulsed Doppler ultrasound; (4) widened diameter of veins; (5) stiff venous wall (membrane); (6) decreased blood flow velocity in the blood vessels, gathered red blood cells, and occurrence of blood flow echoes in the upstream and downstream of the thrombus; and (7) widened collateral vein diameter.

### Statistical analysis:

Data analysis of this study was performed using SPSS 22.0 statistical software. The measurement data conforming to the normal distribution was represented by (), and analyzed by a t-test. Pearson correlation was used to analyze the relationship between the levels of TM, TG, D-D, Fb, CRP and the formation of DVT. The diagnostic value of the above indicators for the formation of DVT after THA was analyzed through the construction of the receiver operating characteristic (ROC) curve. P<0.05 was used to indicate the presence of statistical difference.

## RESULTS

As shown in Table-I, the levels of TM, TG, D-D, Fb and CRP were significantly higher in the study group than that in the control group, with a statistically significant difference between groups (p<0.05). No statistically significant difference was observed in the comparison of the levels of TM, TG, D-D, Fb and CRP between groups before operation (p>0.05). While one day after the operation, the levels of TM, TG, D-D, Fb and CRP were obviously higher in Group-A than those in Group-B, with a statistically significant difference between groups (p<0.05, Table-II).

**Table-I:** Comparison of TM, TG, D-D, Fb and CRP levels between the study group and the control group (*χ̅*±*s*).

Groups	Cases	TM (IU/ml)	TG (mmol/L)	D-D (mg/L)	Fb (g/L)	CRP (mg/L)
Study group	150	10.99±2.35	2.00±0.29	0.78±0.16	3.29±0.51	21.04±3.97
Control group	70	6.31±0.81	1.43±0.18	0.35±0.11	2.37±0.21	7.21±1.25
t		16.202	15.130	20.342	14.515	28.464
P		0.000	0.000	0.000	0.000	0.000

**Table-II:** Comparison of TM, TG, D-D, Fb and CRP levels between Group-A and Group-B before and after the operation (*χ̅*±*s*).

Groups	Cases	TM (IU/ml)		TG (mmol/L)		D-D (mg/L)		Fb (g/L)		CRP (mg/L)	

		Before operation	1d after operation	Before operation	1d after operation	Before operation	1d after operation	Before operation	1d after operation	Before operation	1d after operation
Group-A	46	7.20±1.49	11.89±2.25*	1.95±0.65	2.15±0.32	0.59±0.18	0.81±0.19*	3.01±0.76	3.79±0.42*	31.28±9.65	23.15±4.02*
Group-B	104	7.14±1.54	10.01±2.13*	1.98±0.51	1.70±0.22*	0.57±0.15	0.42±0.11*	2.98±0.85	2.68±0.67*	31.09±9.33	18.21±3.97*
t		0.222	4.899	0.305	9.982	0.707	15.814	0.206	10.361	0.114	7.000
P		0.824	0.000	0.761	0.000	0.481	0.000	0.837	0.000	0.910	0.000

***Note:*** Compared with this Group-Before the operation,

* p<0.05.

**Fig.1:**
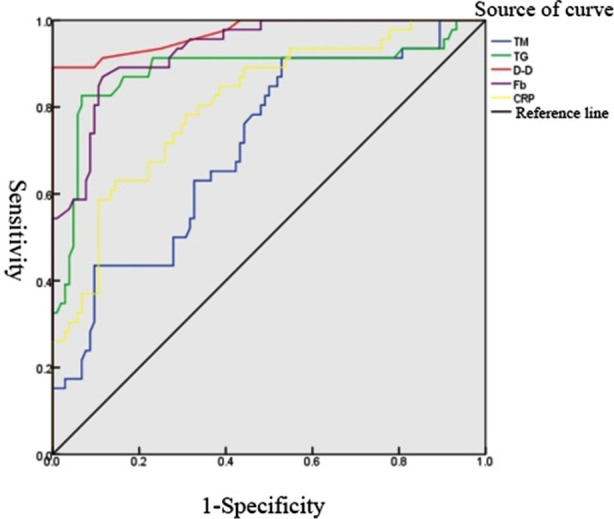
The ROC curve of the diagnosis of DVT formation after THA with TM, TG, D-D, Fb and CRP levels.

According to the results of Pearson correlation analysis (Table-III), the levels of TM, TG, D-D, Fb and CRP were significantly positively correlated with the formation of DVT, showing a statistically significant difference (p<0.05). The diagnostic efficiency results of TM, TG, D-D, Fb, CRP indicators for diagnosing DVT are listed in Table-IV, and the ROC curve is shown in Fig.1.

**Table-III:** Correlation analysis of TM, TG, D-D, Fb and CRP levels with DVT formation.

		DVT	TM	TG	D-D	Fb	CRP
DVT	Pearson correlation	1	0.374**	0.635**	0.793**	0.648**	0.499**
	Significance (two-tailed)		0.000	0.000	0.000	0.000	0.000
	N	150	150	150	150	150	150
TM	Pearson correlation	0.374**	1	0.653**	0.633**	0.621**	0.629**
	Significance (two-tailed)	0.000		0.000	0.000	0.000	0.000
	N	150	150	150	150	150	150
TG	Pearson correlation	0.635**	0.653**	1	0.718**	0.678**	0.674**
	Significance (two-tailed)	0.000	0.000		0.000	0.000	0.000
	N	150	150	150	150	150	150
D-D	Pearson correlation	0.793**	0.633**	0.718**	1	0.728**	0.648**
	Significance (two-tailed)	0.000	0.000	0.000		0.000	0.000
	N	150	150	150	150	150	150
Fb	Pearson correlation	0.648**	0.621**	0.678**	0.728**	1	0.682**
	Significance (two-tailed)	0.000	0.000	0.000	0.000		0.000
	N	150	150	150	150	150	150
CRP	Pearson correlation	0.499**	0.629**	0.674**	0.648**	0.682**	1
	Significance (two-tailed)	0.000	0.000	0.000	0.000	0.000	
	N	150	150	150	150	150	150

** The correlation was significant when the confidence (two-tailed) was 0.01.

**Table-IV:** Diagnostic value of TM, TG, D-D, Fb and CRP levels in DVT formation after THA.

Outcome variables	AUC	SE	P value	95%CI	Cut-off value	Youden’s index	Sensitivity	Specificity
TM	0.709	0.045	0.000	0.620~0.798	9.505	0.384	91.3	47.1
TG	0.882	0.038	0.000	0.807~0.957	1.935	0.759	82.6	93.3
D-D	0.970	0.014	0.000	0.942~0.999	0.595	0.891	89.1	100.0
Fb	0.932	0.020	0.000	0.892~0.972	3.365	0.737	89.1	84.6
CRP	0.807	0.038	0.000	0.732~0.882	22.120	0.486	63.0	85.6

The AUC of the five indicators for diagnosing DVT was 0.709, 0.882, 0.970, 0.932 and 0.807, respectively. According to the optimal critical point, the sensitivity was 91.3%, 82.6%, 89.1%, 89.1% and 63.0%, and the specificity was 47.1%, 93.3%, 100.0%, 84.6% and 85.6% when the value of TM, TG, D-D, Fb and CRP was higher than 9.505 IU/ml, 1.935 mmol/L, 0.595 mg/L, 3.365 g/L and 22.120 mg/L respectively in diagnosing DVT, with statistically significant difference (p<0.05).

## DISCUSSION

Due to traffic problems and changes in people’s living habits, there is an increase in the risk of joint injury, and joint replacement has become one of the important operations clinically. THA has been considered to be the primary surgery for joint replacement, and the complications caused by THA have been concerned by both the departments of medicine and surgery.^9^,^10^ DVT is a common postoperative complication that occurs frequently in the lower limbs, which has common clinical manifestations of limb swelling, pain, skin damage and ulceration, etc. In the absence of timely intervention, it may induce pulmonary embolism and affect the postoperative quality of life.^11^,^12^ The formation of DVT is complex and is a multifactorial process. Its diagnosis may be inaccurate based on clinical symptoms alone.^13^,^14^ Meanwhile, venography is an invasive technique,^15^,^16^ hence it is important to identify more excellent laboratory indicators for its screening.

In our study, the levels of TM, TG, D-D, Fb and CRP in the study group were significantly higher than those in the control group, which were also obviously higher in Group-A than those in Group-B one day after the operation. Besides, the results of correlation analysis revealed that TM, TG, D-D, Fb and CRP were significantly positively correlated with the formation of DVT after THA. As for its potential causes, firstly, TM is related to the occurrence of thrombotic diseases.^17^ It is generally used to predict the damage of vascular endothelial cells, which has an intimate association with coagulation function.

The increase in TM expression may indicate the expansion of the scope of vascular disease and the severity of disease progression. Secondly, the rise in TG level represents the increase of blood lipid level, and the risk of atherosclerosis, vascular blockage and blood flow formation in patients; and the TG level of the healthy is usually lower than 1.70mmol/L.^18^ In this study, the critical value of TG for the diagnosis of DVT was 1.935mmol/L, suggesting that thrombosis was induced by promoting the activity of inflammatory factors when the TG level reached a higher level, leading to a higher risk of DVT. Thirdly, D-D is a fibrin-specific degradation product, and elevation in its expression may indicate thrombosis and dissolution in vivo. It can be regarded as a marker of thrombosis in non-invasive cases.^19^

Fourthly, Fb is a coagulation factor with the highest content in the blood. It is an acute reactive protein with high biological activity,^20^ which is a molecular marker of coagulation and fibrinolysis. The obvious increase of Fb postoperatively may indicate an enhancement in fibrinolysis. Fifthly, elevation in CRP expression may promote the synthesis of coagulation factors, which may potentially lead to a hypercoagulable state in the affected patients, leading to the formation of DVT and the aggravation of the disease.^21^ In the present study, further analysis of the diagnostic efficacy of ROC curve revealed that TM, TG, D-D and Fb had higher sensitivity (>80% in all) to the formation of DVT, yet with relatively reduced diagnostic efficacy of CRP. It can be explained by the reason that enrolled patients undergoing THA might have additional tissue injuries that caused the rise of CRP in varying degrees.

Thus, CRP cannot be used as an independent indicator to diagnose the formation of DVT. Additionally, it may also be related to the smaller sample size of this study and hence was not explored ulteriorly. The conclusions of this study provide clinical reference for the prevention and treatment of deep vein thrombosis in patients after total hip arthroplasty.

### Limitations

It includes small sample size and not being a prospective study design. Our future will be based on the expanded sample size, so as to enhance the scientific significance of this research.

## CONCLUSION

To sum up, findings in our study suggest that the levels of TM, TG, D-D, Fb and CRP are significantly increased in patients with DVT formation after THA. All five indicators may have potential diagnostic value for determining the formation of DVT after THA, which are worthy of clinical reference.

### Authors’ Contributions:

**Hailin Luo:** Designed this study, prepared this manuscript, are responsible and accountable for the accuracy and integrity of the work; **Yong Qiao:** Collected and analyzed clinical data; **Hailin Luo:** Data analysis, Significantly revised this manuscript.
